# How to implement evidence-based practice to prevent and reduce coercion in psychiatry

**DOI:** 10.1192/j.eurpsy.2023.157

**Published:** 2023-07-19

**Authors:** N. Sartorius

**Affiliations:** Association for the Improvement of Mental Health Programmes (AMH), Geneva, Switzerland

## Abstract

**Abstract:**

The UN Convention on the rights of people with disabilities (UN CRPD) raised awareness of the need to find alternatives to coercion in the process of care for people with mental illness and/or mental impairment. In order to promote the application of the CRPD the World Psychiatric Association (WPA) has undertaken to produce a document listing possible alternatives to coercion and proposed to the General Assembly of the WPA to support the recommendations of that document by a WPA Position Statement on the matter.

The presentation will discuss the suggestions included in the Position Statement and in the review of options to reduce coercion in the WPA materials.

**Disclosure of Interest:**

None Declared

